# Validation and cultural translation for the Brazilian Portuguese version of the Estro-Androgenic- Symptom Questionnaire in Women

**DOI:** 10.61622/rbgo/2024rbgo56

**Published:** 2024-07-26

**Authors:** Cássia Raquel Teatin Juliato, Ana Aline Coelho Oswaldo, Camila Carvalho de Araújo, Marina Rotoli, Lúcia Costa-Paiva, Rossella Nappi, Luiz Gustavo Oliveira Brito

**Affiliations:** 1 Department of Obstetrics and Gynecology School of Medical Sciences Universidade Estadual de Campinas Campinas SP Brazil Department of Obstetrics and Gynecology, School of Medical Sciences, Universidade Estadual de Campinas, Campinas, SP, Brazil.; 2 Medical School Faculdade São Leopoldo Mandic Campinas SP Brazil Medical School, Faculdade São Leopoldo Mandic, Campinas, SP, Brazil.; 3 Department of Clinical, Surgical, Diagnostic and Pediatric Sciences University of Pavia Pavia Italy Department of Clinical, Surgical, Diagnostic and Pediatric Sciences, University of Pavia, Pavia, Italy.; 4 Research Center for Reproductive Medicine, Gynecological Endocrinology and Menopause IRCCS Policlinico S. Matteo Pavia Italy Research Center for Reproductive Medicine, Gynecological Endocrinology and Menopause, IRCCS Policlinico S. Matteo, Pavia, Italy.

**Keywords:** EASQ-W, Menopause, Estrogen, Androgen, Signs and symptoms, Surveys and questionnaires, Validation studies

## Abstract

**Objective:**

This study aimed to translate and validate the Estro-Androgenic-Symptom Questionnaire in Women (EASQ-W) into Brazilian Portuguese language, as we hypothesized that this tool would be consistent for addressing the specific context of hormonal symptoms in menopause.

**Methods:**

In a cross-sectional study, a total of 119 women with Genitourinary Syndrome of Menopause (GSM) and 119 climacteric women without GSM were included. The EASQ-W was translated, and its psychometric properties were rigorously examined. Participants completed questionnaires covering sociodemographic details, the EASQ-W, and the Menopause Rating Scale (MRS). A subgroup of 173 women was re-invited after 4 weeks for test-retest analysis of the EASQ-W. Additionally, the responsiveness of the questionnaire was evaluated in 30 women who underwent oral hormonal treatment.

**Results:**

The internal consistency of the EASQ-W was found to be satisfactory in both GSM and control groups (Cronbach’s alpha ≥ 0.70). Notably, a floor effect was observed in both groups; however, a ceiling effect was only evident in the sexual domain of the GSM group. Construct validity was established by comparing the EASQ-W with the MRS, yielding statistically significant correlations (0.33831-0.64580, p < 0.001). The test-retest reliability over a 4-week period was demonstrated to be satisfactory in both the GSM and control groups (ICC 0.787-0.977). Furthermore, the EASQ-W exhibited appropriate responsiveness to oral hormonal treatment (p < 0.001).

**Conclusion:**

This study successfully translated and validated the Estro-Androgenic-Symptom Questionnaire in Women (EASQ-W) into Brazilian Portuguese, with satisfactory internal consistency, test-retest reliability, and construct validity.

## Introduction

Menopause signifies a significant juncture in a woman’s life, marked by a cascade of physiological and psychological transformations. As life expectancy continues to rise, it is projected that approximately one-third of a woman’s lifespan will be experienced in the postmenopausal period.^(1)^The array of prevalent menopausal symptoms encompasses vasomotor manifestations, including hot flashes and night sweats, afflicting 57% of women, sexual dysfunction affecting 60%, and joint discomfort experienced by 62%.^(2)^In tandem, psychological symptoms such as irritability, anxiety, sleep disturbances, and urinary issues^(3)^ often accompany this life stage.

A distinctive facet of menopausal experience, the genitourinary syndrome of menopause (GSM), is characterized by a constellation of symptoms encompassing vaginal dryness, burning sensations, reduced lubrication, and other manifestations within the female genitourinary domain. This syndrome arises from histological, anatomical, and clinical transformations attributed to diminishing ovarian hormones.^(4-6)^Unlike the transient nature of vasomotor symptoms, GSM symptoms frequently exhibit an escalating trajectory over time,^(7)^profoundly affecting women’s quality of life and impinging on their sexual well-being.^(8)^The prevalence of GSM symptoms is notable, with approximately 55% of women reporting vaginal dryness, 44% encountering dyspareunia, 37% experiencing vulvar irritation, and 15% grappling with urinary incontinence.^(9)^

Within this dynamic, androgens assume a pivotal role in a woman’s existence. The life course of androgen production entails a natural decline, a phenomenon that becomes more evident over time. Despite estrogen decline, testosterone levels tend to stabilize, with studies indicating that the ovaries of menopausal women sustain a consistent testosterone output.^(10)^However, age-related factors translate to a diminution in androgen production, with serum DHEA and S-DHEA levels in women of their eighth decade plummeting to about a fifth of levels observed in their third decade.^(11,12)^This phenomenon has led to the conceptualization of androgen deficiency syndrome, denoting a reduction in androgen production post-menopause, giving rise to symptoms encompassing reduced well-being, dysphoric mood, fatigue, and alterations in sexual function.^(13)^

In the realm of clinical research, validated questionnaires serve as indispensable instruments. Regrettably, a dearth of questionnaires exists that comprehensively assess symptoms of estrogen, androgen deficiency, and GSM in peri- and post-menopausal women. Notably, the Estro-Androgenic Symptom Questionnaire in Women^(14)^ was developed and validated to offer enhanced characterization of midlife women experiencing vulvovaginal atrophy and GSM. Motivated by this context, the present study seeks to bridge the gap by translating and culturally validating the EASQ-W questionnaire into Brazilian Portuguese, facilitating its application and utility within this population.

## Methods

We conducted a cross-sectional study spanning from June 2022 to July 2023 at the Department of Obstetrics and Gynecology, School of Medical Sciences, University of Campinas (UNICAMP). This investigation adhered to the guidelines for Reporting Reliability and Agreement Studies (GRRAS) concerning projects involving psychometric variables.^(15)^Approval from the Institutional Review Board was obtained, and all participating women provided informed consent after a comprehensive explanation of the study objectives.

We included 119 menopausal women exhibiting vasomotor symptoms (e.g., hot flashes, night sweats), genital atrophy, vaginal dryness, urgency, urinary incontinence, sexual issues (such as reduced libido, orgasmic dysfunction), psychological symptoms (e.g., irritability, discouragement, anxiety, cognitive decline, concentration difficulties, insomnia), and muscle or joint discomfort (group: menopause). Hormone therapy was individualized according to the needs and characteristics of the patient, with continuous combined therapy of low dose estradiol (1 mg oral or 0.75 mg transdermal) associated with to an oral progestogen (norethisterone 0.5 mg or micronized progesterone 100 mg) for women with a uterus or oral or transdermal isolated estrogen only for hysterectomized women. The control group (n=119) encompassed women with regular menstrual cycles (frequency between 28-38 days)^(16)^and devoid of menopausal symptoms. In totality, 238 women were included in the study. Exclusion criteria encompassed women undergoing menopausal symptom treatment (systemic or vaginal hormone therapy with estrogens, progestogens, or androgens), pregnant women, and individuals with reading and/or comprehension impediments.

Following authorization from the questionnaire author (R.E.N.), a meticulous translation process was undertaken. Two native Brazilian translators, proficient in English, translated the questionnaire from English to Brazilian Portuguese, with official authorization for scientific document translation. The synthesis of the translations yielded a common version, which was subsequently back translated into English by a third translator. Concordance between the back-translated instrument and the original version was verified. The Brazilian Portuguese EASQ-W was evaluated by an expert committee (C.R.T.J., L.G.O.B., C.C.A.) for linguistic adaptation to enhance participant comprehension. A pre-test involving an interview-based application of the EASQ-W to 30 women revealed no necessity for further adjustments, leading to approval of the final questionnaire for research use (Supplementary Material - Table 1S).

Before applying the EASQ-W, we collected baseline variables as age, body mass index (BMI), ethnics, parity, comorbidities, menopausal status, and sexual activity. Psychometric variables for analyzing the EASQ-W were internal consistency, floor and/or ceiling effect, test-retest reliability, responsiveness to treatment, and discriminant/construct validity.

The EASQ-W assesses age-related, psychological, anatomical, vasomotor, urogenital, weight gain, and sleep-related symptoms, therefore offering both researchers an easy and reliable method to quantify relative and absolute change in symptom frequency after treatment. The EASQ-W includes the prevalence of symptoms (vasomotor, psychological symptoms, insomnia, muscle and joint discomfort, sexual function, and urogenital symptoms), in addition to being an easy-to-understand and self-administered questionnaire. It contains 32 questions, which are grouped into eight domains: age, psychological, sexual, anatomical, weight, urological, vasomotor symptoms, and sleep. Each question can be answered with “no” or “yes”, and in the case of “yes” a score of 1-10 must be assigned. The total score is obtained by the sum of all scores.

The MRS has 11 questions divided into three domains: somatic-vegetative symptoms, psychological symptoms, and urogenital symptoms. The response to each question is rated on an intensity scale ranging from zero (no symptoms) to four (very intense symptoms). The total MRS score is obtained by adding the scores for each domain, so that the higher the score, the more intense the symptoms and the worse the quality of life. These items are categorized into the following 3 subscales or domains: somatic-vegetative (4 items), psychological (4 items), and urogenital (3 items).

Descriptive statistics entailed frequency tables with percentages for categorical variables and mean and standard deviation for numerical variables. Comparative analysis involved Chi-square or Fisher’s exact tests for categorical variables and the non-parametric Mann-Whitney test for numerical variables. Internal consistency was evaluated using Cronbach’s alpha index, with values of 0.70-0.95 deemed adequate. Test-retest reliability was assessed using mean difference and intraclass correlation coefficient (ICC). Construct validity involved correlation testing between EASQ-W and MRS scores. Floor and ceiling effects were determined by identifying scores exceeding 15% below or above the end value. Responsiveness was assessed through a pre- and post-intervention EASQ-W score comparison using the Wilcoxon test.

The EASQ-W score cutoff point distinguishing case and control groups was obtained via ROC curve analysis. A significance level of 5% was applied, and SAS statistical package (Cary, NC, USA) was utilized for analyses.

The study complied with Brazilian ethical standards involving human beings protocol 4.814.688 (CAAE: 45451521.8.0000.5404).

## Results

### Baseline characteristics

Table 1 illustrates the distribution of the two study groups based on sociodemographic and obstetric variables. Notably, patients within the Genitourinary Syndrome of Menopause (GSM) group exhibited distinct characteristics compared to the control group, predominantly consisting of women in their reproductive phase. Specifically, individuals in the GSM group were older than those in the control group (32.2 ± 10.4 years versus 57.4 ± 10.9 years, p < 0.001). Additionally, they displayed a lower educational attainment (p < 0.001) and a heightened prevalence of various comorbidities, including arterial hypertension (p < 0.001), diabetes mellitus (p < 0.001), and urinary incontinence (p < 0.001). However, no statistically significant differences were observed in terms of parity, body mass index, ethnicity, smoking habits, and the presence of chronic obstructive pulmonary disease between the two groups. This comprehensive examination of sociodemographic and obstetric variables underscores the distinct profiles of the GSM and control groups, which is instrumental for contextualizing the subsequent findings of the study.

**Table 1:** Baseline characteristics of the women included in the two study groups (n=238)

Variable	GSM Group (n= 119)	Control Group (n= 119)	p-value
Age years (X±SD)	57.4±10.9	32.2±10.4	<0.001*
BMI (X±SD)	26.5±5.6	27.49±4.8	0.08*
Schooling years (X±SD)	12.8±9.1	16.9±5.5	<0.001*
Ethnicity n (%) n=231			
white	78 (68.4)	75 (59.1)	0.994
Not white	36 (31.6)	52 (40.9)	
Pregnancy	2.8±1.9	1.1±1.3	<0.001
Parity			
vaginal	1.6±1.8	1.07±1.5	0.09*
Cesarean section	0.8±1	1.02±1.1	0.82*
Sexually Active (yes)	70 (59.3)	110 (92.4)	<0.001
Hypertension (yes) n=237	57 (48.3)	20 (16.83)	<0.001**
Diabetes Mellitus (yes) n=236	21 (18)	3 (2.5)	<0.001**
COPD (Yes)	4 (3.3)	4 (3.3)	1***
Smoking(yes) n=224	10 (8.5)	11 (610.4)	0.626**
Urinary incontinence (yes)	66 (55.5)	22 (18.5)	<0.001**

GSM - Genitourinary Syndrome of Menopause; BMI - body mass index; COPD - severe obstructive pulmonary disease; X - mean; SD - standard deviation; *p-value; ** Chi square; ***Fisher’s exact test

### Discriminant validity and floor/ceiling effects of EASQ-W and MRS scores

Table 2 presents the outcomes of the EASQ-W and Menopause Rating Scale (MRS) questionnaires among women belonging to the Genitourinary Syndrome of Menopause (GSM) and control groups. Our analysis reveals a substantial contrast in the scores across all domains of the EASQ-W questionnaire between these groups, except for the psychological domain. This noteworthy exception underscores the questionnaire’s excellent discriminant validity, as it effectively discerns between the two distinct groups based on the presence of genitourinary symptoms associated with menopause.

**Table 2:** Discriminant validity of the EASQ-W and Menopause Rate Scale scores between women with and without genitourinary syndrome of menopause

	SGM (n=119)			Control (n= 119 )			p-Value*
	M±DP	Minimum	Maximum	M±DP	Minimum	Maximum	
EASQ-W							
Age	33.8±19.2	0	48	13.6 ± 16.6	0	73	<0.001
Psychological	16±13.4	0	27	11.5±11.7	0	50	0.013
Sexual	14.8±9.8	0	23	5.9±8.9	0	30	<0.001
Anatomical	16±12.4	0	25	3.3±6.6	0	31	<0.001
Body weight	11.2±10	0	20	6±07	0	30	<0.001
Urological	13.2±10.2	0	22	5.5±8.4	0	30	<0.001
Vasomotor	6.5±7	0	10	1.6±3.6	0	18	<0.001
Sleep	9.8±8.4	0	16	6.1±7.4	0	27	<0.001
Total	120.6±64.8	0	169	53.61±55.9	0	254	<0.001
MRS							
Somatovegetative	5.5±3.9	0	15	2.65±3	0	11	<0.001
Psychological	5.2±4.7	0	16	5.5±4.4	0	16	0.525
Urogenital	4.4±3.2	0	12	1.7±2.8	0	12	<0.001
Total	15.2±9.9	0	35	9.8±8.7	0	35	<0.001

GSM - genitourinary menopausal syndrome; M - mean; SD - standard deviation; MRS - menopause rate scale questionnaire

Regarding to floor and ceiling effects, control group showed a floor effect in all EASQ-W domains. Women with GSM showed a floor effect in all domains and a floor and ceiling effect in the sexual domain (Table 3).

**Table 3:** Ceiling and floor effect analysis of questionnaire scores EASQ-W

EASQ-W	GSM Group		Control Group	
	Frequency	Percent	Frequency	Percent
Age				
Floor 0-12	21	17.6 (Floor effect)	72	60.5 (Floor Effect)
13-67	96	89.7	46	38.7
Ceiling 68-80	2	1.7	1	0.8
Psychological				
Floor 0-7	41	34.5 (Floor effect)	53	44.5 (Floor Effect)
8-42	74	62.2	63	52.9
Ceiling 43-50	4	0.3	3	2.6
Sexual				
Floor 0-4	22	18.5(Floor effect)	72	60.5 (Foor Effect)
5-25	77	64.7	39	32.8
Ceiling 26-30	20	16.8 (Ceiling effect)	8	6.7
Anatomical				
Floor 0-7	37	31.1 (Floor effect)	100	84.3 (Floor Effect)
8-42	79	66.4		
Ceiling 43-50	3	2.5	19	15.7
Body Weight				
Floor 0-4	38	31.9 (Floor effect)	64	53.8 (Floor effect)
5-25	66	55.4	52	43.7
Ceiling 26-30	15	12.7	3	2.5
Urological				
Floor 0-4	33	27.7 (Floor effect)	79	66.4 (Floor effect)
5-25	69	58	36	30.3
Ceiling 26-30	17	14.3	4	3.3
Vasomotor				
Floor 0-3	54	46.2 (Floor effect)	99	83.2(Floor effect)
4-16	47	40.2	19	16
Ceiling 17-20	16	13.6	1	0.8
Sleep				
Floor 0-4	36	30.3 (Floor effect)	61	51.3 (Floor effect)
5-25	76	63.9	53	44.5
Ceiling 26-30	7	5.8	5	4.2
Total				
Floor 0-48	21	17.6 (Floor effect)	61	51.3 (Floor effect)
49-271	97	81.5	53	44.5
Ceiling 272-320	1	0.9	5	4.2

Internal consistency of the EASQ-W questionnaire can be evaluated in table 4. Cronbach’s Alpha values show the reliability of the questionnaire, that is, how much the partial measures are consistent with each other. The anatomical, vasomotor and sleep domains showed values below 0.70, but above 0.5, which shows good agreement. The other domains showed better agreement, with values above 0.70.

**Table 4:** Internal consistency of the EASQ-W questionnaire by study group

EASQ-W	GSM Group (n=119)	Control Group (n=119)
	Cronbach’s alpha	Cronbach’s alpha
Age	0.822	0.860
Psychological	0.793	0.829
Sexual	0.798	0.896
Anatomical	0.697	0.690
Body Weight	0.804	0.615
Urological	0.809	0.868
Vasomotor	0.687	0.563
Sleep	0.593	0.643
Total	0.921	0.945

### Test–retest reliability, construct validity, and responsiveness to treatment

**Test-Retest Analysis:** Following a 4-week interval, a subset of 173 women (106 from the GSM group and 67 from the control group) were randomly selected for test-retest assessment. During this retest, the questionnaire under investigation was administered. Evaluation of the test-retest data revealed minimal variation across most domains. Notably, the intraclass coefficient (ICC) exhibited values approximating 1, indicating a high level of correlation between the responses during the initial test and the subsequent retest. This outcome underscores the excellent consistency in participant responses over time (Table 5).

**Table 5:** Test and retest reliability of the EASQ-W questionnaire

EASQ-W	Test Scores	Retest score	p-value*	Difference	ICC	95%
	X ±SD	X ±SD		X ±SD		
GSM group (n=106)						
Age	35±19	36.3±20.2	<0.001	1.25±4	0.977	0.964-0.985
Psychological	16.2±13.4	16.7±13.4	0.041	0.48±4.4	0.945	0.921-0.962
Sexual	15.6±9.7	15.9±10.1	0.086	0.31±2.9	0.958	0.939-0.971
Anatomical	16.2±12.6	15.9±10.1	<0.001	0.6±2.6	0.979	0.968-0.978
Body Weight	11.3±10.2	11.5±10.3	0.211	0.26±2.6	0.968	0.954-0.978
Urological	13.5±10.3	13.8±10.4	0.258	0.23±3.1	0.956	0.936-0.970
Vasomotor	6.6±7.1	7.1±7.3	0.023	0.42±3.4	0.892	0.846-0.925
Sleep	10±8.7	9.9±8.4	0.941	-0.10±2.9	0.944	0.919-0.962
Total	124.9±64.7	128.4±67.3	<0.001	3.4±12	0.982	0.973-0.988
Control group (n=67)						
Age	12.2±16.2	10.8±15.4	0.019	-1.5±5.1	0.945	0.908-0.966
Psychological	10.8±11.1	9.5±11	0.020	-1.4±4.6	0.908	0.849-0.944
Sexual	5.5±9	5.6±9.2	0.423	0.15±2.2	0.972	0.955-0.982
Anatomical	2.4±6	2.4±6	0.406	0.01±0.7	0.972	0.955-0.983
Body weight	4.9±5.8	4±6.1	0.062	-0.9±3.4	0.787	0.673-0.864
Urological	4.7±8	4.6±8.3	0.870	-0.12±2.6	0.870	0.919-0.968
Vasomotors	1.5±3.2	1.5±3.1	0.938	0.03±1.1	0.936	0.898-0.960
Sleep	5.6±7.5	5.4±7.4	0.622	-0.18±1.3	0.985	0.977-0.991
*Total*	47.6±52.6	43.8±53.3	0.141	-3.8±12.7	0.969	0.948-0.981

ICC - intraclass coefficient; SD - standard deviation; *p-value between test and retest (Wilcoxon); **p ICC value

**Construct Validity:** Construct validity was established by comparing the scores of the EASQ-W with those of the Menopause Rating Scale (MRS). This analysis yielded a moderate correlation between the two questionnaires, with a correlation coefficient (r) of 0.64580 (p < 0.001), as depicted in table 6. This finding substantiates the congruence between the EASQ-W and MRS scores in evaluating menopausal symptoms.

**Table 6:** Construct validity between EASQ-W and MRS questionnaires

EASQ-W	MRS Somatic	MRS Psychological	MRS Urogenital	MRS Total
Age	r=0.56628 p<0.001	r=0.52308 p<0.001	r=0.53708 p<0.001	r=0.61657 p<0.001
Psychological	r=0.48301 p<0.001	r=0.54563 p<0.001	r=0.49810 p<0.001	r=0.57943 p<0.001
Sexual	r=0.33831 p=0.0002	r=0.36364 p<0.001	r=0.61735 p<0.001	r=0.46331 p<0.001
Anatomical	r=0.44661 p=0.0002	r=0.343104 p=0.0002	r=0.57002 p<0.001	r=0.54541 p<0.001
Body Weight	r=0.34013 p=0.0002	r=0.30247 p=0.0008	r=0.33190 p=0.0002	r=0.34402 p=0.0002
Urological	r=0.35050 p<0.0001	r=0.32401 p=0.0003	r=0.40212 p<0.0001	r=0.45851 p<0.0001
Vasomotor	r=0.28466 p=0.0017	r=0.28700 p=0.0016	r=0.28466 p=0.0017	r=0.31793 p=0.0004
Sleep	r=0.40329 p<0.001	r=0.36989 p<0.001	r=0.37929 p<0.001	r=0.44199 p<0.001
Total	r=0.55068 p<0.001	r=0.55900 p<0.001	r=0.60322 p<0.001	r=0.64580 p<0.001

r - Spearman correlation; p-value

**Responsiveness Assessment:** To gauge responsiveness, a subgroup of women (n=30) from the GSM group underwent a 90-day regimen of oral hormonal therapy for menopause. Subsequent analysis of the scores across all domains and the total score of the EASQ-W demonstrated a noteworthy reduction post-treatment, indicating a favorable response to the therapy. Detailed results are presented in table 7.

**Table 7:** Responsiveness after 90 days of oral hormonal therapy (n= 30)

EASQ-W (X±SD)	Pre-treatment	Post treatment	p-value*
Age	42.3 (17.7)	13.6 (18.1)	<0.001
Psychological	23.9 (12.3)	7.3 (11.1)	<0.001
Sexual	18.6 (9.3)	5.3 (7.5)	<0.001
Anatomical	22.3 (12.3)	7.3 (10.7)	<0.001
Body Weight	13.6 (11.8)	4.6 (8.4)	<0.001
Urological	15.2 (9.6)	4.7 (6.6)	<0.001
Vasomotor	11.9 (7.7)	7.1 (6.6)	<0.001
Sleep	12.9 (7.7)	4.1 (6.8)	<0.001
Total	160.7 (57.2)	51.1 (68.9)	<0.001

*Wilcoxon test

**Figure 1:**
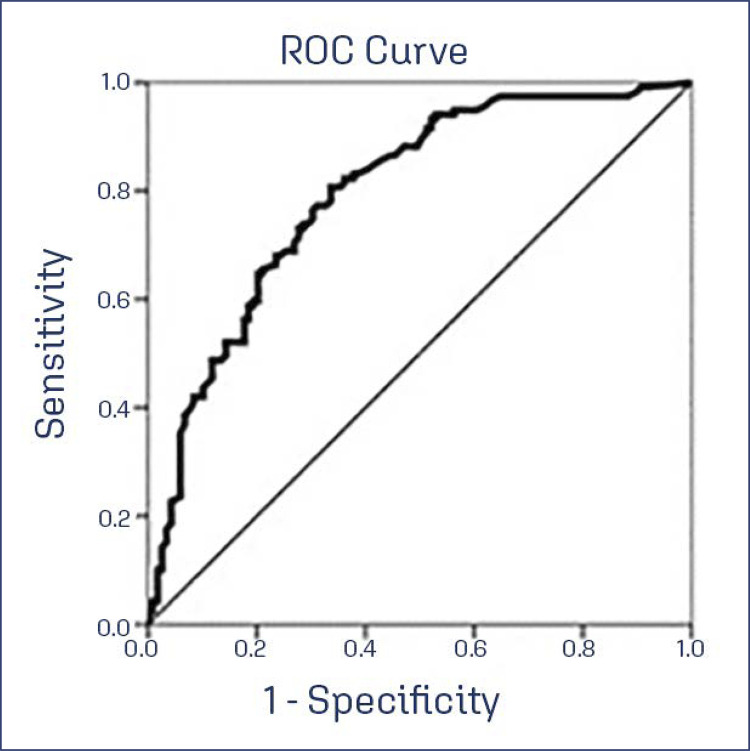
ROC curve

**ROC Curve Analysis and Cut-off Point Determination:** A Receiver Operating Characteristic (ROC) curve was constructed to ascertain the questionnaire’s cut-off point capable of effectively distinguishing between women with and without GSM. The analysis indicated that a cut-off point exceeding 60 points achieved a sensitivity of 80% (95% CI: 72.2 - 80.1), specificity of 66.4% (95% CI: 57.7 - 74.6), positive predictive value of 70.6% (95% CI: 62.1 - 84.9), and a negative predictive value of 77.5% (95% CI: 67.9 - 84.9) for identifying GSM. This information, depicted in figure 1, underscores the utility of the EASQ-W in effectively discriminating between individuals with and without GSM.

## Discussion

This study successfully accomplished the cultural translation and psychometric evaluation of the Estro-Androgenic Symptom Questionnaire in Women (EASQ-W) for Brazilian Portuguese. The EASQ-W, a comprehensive 32-item questionnaire encompassing key symptoms associated with estrogen and androgen decline following menopause, was meticulously translated, and culturally validated to ensure its relevance and reliability within this population. Notably, this approach addresses a significant gap in the existing literature, as few questionnaires adopt this comprehensive perspective.

Distinct from vasomotor symptoms, the Genitourinary Syndrome of Menopause (GSM) precipitates chronic histological, anatomical, and clinical changes in the genital and lower urinary tract, attributed to the gradual reduction of ovarian hormones.^(4-6)^This syndrome’s progressive nature underscores the urgency of accurate diagnosis and intervention, especially given the potential for worsening symptoms and complications such as vaginal stenosis.^(1,17,18)^ In light of this complexity and the demand for more advanced survey tools, the translation and cultural validation of the EASQ-W were undertaken.

The psychometric evaluation of the EASQ-W revealed its strong cultural adequacy. Its domain and total scores exhibited significant differences between the case and control groups, indicative of robust discriminant validity. While the psychological domain exhibited no variance between the groups, this can be attributed to the domain’s broader nature encompassing psychological concerns linked to both GSM and symptoms arising from estrogen and androgen decline. Internal consistency, assessed using the Cronbach index, indicated favorable results, with values exceeding 0.70, indicating strong internal coherence. Although the anatomical, vasomotor, and sleep domains presented slightly lower values, their adequacy was still maintained. The test-retest reliability analysis demonstrated the questionnaire’s stability over time, with consistent results after a 4-week interval.

Furthermore, the EASQ-W exhibited a notable and moderate correlation with the Menopause Rating Scale (MRS), which assesses climacteric symptoms and quality of life. Both questionnaires effectively detected postmenopausal syndrome symptoms, with the EASQ-W distinguishing itself by its broader scope and domain-specific questions. The MRS is an excellent and well-known questionnaire applied in clinical studies, but it contains less detail on symptoms and few questions. Some symptoms of menopause overlap with age-related complaints, particularly complaints related to androgen deficiency such as decreased strength, memory problems, decreased energy, signs of aging. The EASQ-W also addresses some more detailed questions about psychological aspects not covered in the MRS such as fear, panic attacks, pleasure of living. One of the main differences in the application of this questionnaire is the detailing of urogenital symptoms, which contains five specific questions about vaginal symptoms and three about urinary incontinence/urinary symptoms. Despite being longer and requiring more interview time, this detail of the EASQ-W can be advantageous when one needs a more adequate characterization of the symptoms arising not only from menopausal hypoestrogenism, but also from factors related to aging and the drop in androgenic levels that is observed with the advancement of age and during post-menopause.

Assessment of the EASQ-W’s responsiveness following treatment underscored its potential as a valuable tool for monitoring treatment outcomes, evidenced by the significant reduction in scores observed 30 days post-treatment. The study also established a cut-off point of 60 for diagnosing GSM and symptoms linked to estrogen and androgen decline, an invaluable addition that was not present in the original version of the questionnaire.

This study’s strengths encompass a robust sample size, comprehensive evaluation of the most important psychometric variables (discriminant and construct validity, ceiling and floor effect, internal consistency, test/retest reliability, responsiveness) into a single study, establishment of a cut-off point for EASQ-W questionnaire, and assessment of responsiveness to pharmacological treatment. However, some baseline differences between the study groups represent a limitation.

## Conclusion

In summation, the EASQ-W stands as a valid instrument, adept at discriminating symptoms arising from hormonal decline post-menopause. Its commendable internal consistency, reliability, and responsiveness render it a valuable tool for clinical assessment and treatment evaluation. The newly established cut-off point of 60 contributes to its diagnostic utility for identifying postmenopausal syndrome.

## Supplementary Material

**Table 1:** . EASQ-W questionnaire in Brazilian Portuguese language

EASQ-W Questionário sobre sintomas [de insuficiência] estro-androgênica em mulheres
Instruções: Coloque um “X” sobre a resposta que ·melhor descreve como você se sentiu nas últimas 4 semanas. Se a resposta à pergunta é S~ indique com um número de 1 a 10 a intensidade que atribui ao sintoma/problema; isto é; quanto isso pesa em uma escala de valores (por exemplo, não o número de ondas de calor ou quilos de peso corpóreo, mas como você se sente, de 1 a 10, em relação àquele sintoma/problema).
1. Notou o aparecimento de secura interna e/ou secura externa na vagina? Sim ( ) Não ( ) Se Sim ... De l a 10, indique um número ____.
2. Você notou alguma perda de energia/vitalidade? Sim ( ) Nã0 ( ) Se Sim ... De 1 a 10, indique um número ____ .
3. Notou uma diminuição no tônus/força muscular e na resistência física? Sim ( ) Não ( ) Se Sim ... De l a l0, indique um número ____.
4. Teve a impressão de sua altura. estar diminuindo? Sim ( ) Não ( ) Se Sim ... De l a l0, indique um Número_____ .
5. Notou uma diminuição do “prazer de viver”? Sim ( ) Não ( ) Se Sim ... De 1 a 10, indique um número______.
6. Você se sentiu mais triste e/ou com pensamentos/medos estranhos? Sim ( ) Não ( ) Se Sim ... De 1 a 10, indique um número_____ .
7. Recentemente, teve crises verdadeiras de pânico? Sim ( ) Não ( ) Se Sim ... De 1 a 10, indique um número____ .
8. Você se sentiu mais ansiosa e/ou irritável? Sim ( ) Não ( ) Se Sim ... De 1 a 10, indique um número ____.
9. Observou o aparecimento de rubor diurno e/ou noturno? Sim ( ) Não ( ) Se Sim ... De 1 a 10, indique um número____.
10. Observou o surgimento de suores diurnos e noturnos? Sim ( ) Não ( ) Se Sim ... De 1 a 10, indique um número ____ .
11. Observou o aparecimento de palpitações/frequência cardíaca aumentada? Sim ( ) Não ( ) Se Sim ... De 1 a 10, indique um número______ .
12. Notou que sofre mais de dor de cabeça? Sim ( ) Não ( ) Se Sim ... De 1 a 10, indique um número_____.
13. Observou uma diminuição no desejo/nas fantasias sexuais? Sim ( ) Não ( ) Se Sim ... De 1 a 10, indique uni número _____ .
14. Percebeu diminuição em sua excitação/seu prazer sexual? Sim ( ) Não ( ) Se Sim ... De 1 a 10, indique um número _____ .
15. Teve a impressão de que seus órgãos genitais externos encolheram? Sim ( ) Não ( ) Se Sim ... De 1 a 10, indique um número ____ .
16. Percebeu que sua lubrificação está reduzida a ponto de sentir dor nas relações sexuais? Sim ( ) Não ( ) Se Sim ... De 1 a 10, indique um número ____ .
17. Teve a impressão de que sua vagina está menor do que era antes? Sim ( ) Não ( ) Se Sim ... De 1 a 10, indique um número _____.
18. Reduziu a frequência de suas relações sexuais? Sim ( ) Não ( ) Se Sim ... De 1 a 10, indique um número _____ .
19. Sentiu que parece urinar com mais frequência do que antes/com mais urgência? Sim ( ) Não ( ) Se Sim ... De 1 a 10, indique um número .____ .
20. Acordou mais durante a noite, com urgência para urinar? Sim ( ) Não ( ) Se Sim ... De 1 a 10, indique um número_____.
21. Percebeu que segura menos sua urina do que antes, quando faz algum esforço? Sim ( ) Não ( ) Se Sim ... De 1 a 10, indique um número_____.
22. Observou recentemente queda/diminuição dos pelos pubianos/axilares? Sim ( ) Não ( ) Se Sim ... De 1 a 10, indique um número _____ .
23. Adormeceu depois do jantar mais vezes do que anteriormente? Sim ( ) Não ( ) Se Sim ... De 1 a 10, indique um número ____ .
24. Acordou antes de seu despertador matinal tocar? Sim ( ) Não ( ) Se Sim ... De 1 a 10, indique um número____.
25. Observou recentemente uma deterioração no desempenho do seu trabalho? Sim ( ) Não ( ) Se Sim ... De 1 a 10, indique um número____.
26. Tem mais lapsos de memória/confusão do que antes? Sim ( ) Não ( ) Se Sim ... De 1 a 10, indique um número_____.
27. Sentiu um aumento de dor nos ossos e articulações? Sim ( ) Não ( ) Se Sim ... De 1 a 10, indique um número ____ .
28. Sentiu que sua barriga aumentou mais do que antes? Sim ( ) Não ( ) Se Sim ... De 1 a l0, indique um número_____ .
29. Ganhou peso recentemente? Sim ( ) Não ( ) Se Sim ... De 1a l0, indique um número _____ .
30. Percebe mais rugas/sinais de envelhecimento no espelho? Sim ( ) Não ( ) Se Sim ... De 1 a 10, indique um número _____ .
31. Sentiu que tinha mais apetite/mais ataques de fome? Sim ( ) Não ( ) Se Sim ... De 1 a 10, indique um número_____ .
32. Sentiu que tinha uma qualidade de vida mais baixa, de modo geral? Sim ( ) Não ( ) Se Sim ... De 1 a 10, indique um número____.

## References

[1] The NAMS 2020 GSM Position Statement Editorial Panel (2020). The 2020 genitourinary syndrome of menopause position statement of The North American Menopause Society. Menopause.

[2] Makara-Studzinska MT, Krys-Noszczyk KM, Jakiel G (2014). Epidemiology of the symptoms of menopause - an intercontinental review. Prz Menopauzalny.

[3] Lui JF, Baccaro LF, Fernandes T, Conde DM, Costa-Paiva L, Pinto AM (2015). Factors associated with menopausal symptoms in women from a metropolitan region in Southeastern Brazil: a population-based household survey. Rev Bras Ginecol Obstet.

[4] Portman DJ, Gass ML, Vulvovaginal Atrophy Terminology Consensus Conference Panel (2014). Genitourinary syndrome of menopause: new terminology for vulvovaginal atrophy from the International Society for the Study of Women's Sexual Health and the North American Menopause Society. J Sex Med.

[5] Daan NM, Fauser BC (2015). Menopause prediction and potential implications. Maturitas.

[6] Portman DJ, Gass ML, Vulvovaginal Atrophy Terminology Consensus Conference Panel (2014). Genitourinary syndrome of menopause: new terminology for vulvovaginal atrophy from the International Society for the Study of Women's Sexual Health and the North American Menopause Society. Menopause.

[7] DiBoaventura M, Luo X, Moffat M, Bushmakin AG, Kumar M, Bobula J (2015). The association between vulvovaginal atrophy symptoms and quality of life among postmenopausal women in the United States and Western Europe. J Womens Health (Larchmt).

[8] Palacios S, Mejia A, Neyro JL (2015). Treatment of the genitourinary syndrome of menopause. Climacteric.

[9] Wierman ME, Arlt W, Basson R, Davis SR, Miller KK, Murad MH (2014). Androgen therapy in women: a reappraisal: an Endocrine Society clinical practice guideline. J Clin Endocrinol Metab.

[10] Braunstein GD, Reitz RE, Buch A, Schnell D, Caulfield MP (2011). Testosterone reference ranges in normally cycling healthy premenopausal women. J Sex Med.

[11] Davison SL, Bell R, Donath S, Montalto JG, Davis SR (2005). Androgen levels in adult females: changes with age, menopause, and oophorectomy. J Clin Endocrinol Metab.

[12] Baulieu EE, Thomas G, Legrain S, Lahlou N, Roger M, Debuire B (2000). Dehydroepiandrosterone (DHEA), DHEA sulfate, and aging: contribution of the DHEAge Study to a sociobiomedical issue. Proc Natl Acad Sci U S A.

[13] Bachmann G, Bancroft J, Braunstein G, Burger H, Davis S, Dennerstein L (2002). Female androgen insufficiency: the Princeton consensus statement on definition, classification, and assessment. Fertil Steril.

[14] Nappi RE, Di Carlo C, Cucinella L, Gambacciani M (2020). Viewing symptoms associated with Vulvovaginal Atrophy (VVA)/Genitourinary syndrome of menopause (GSM) through the estro-androgenic lens - Cluster analysis of a web-based Italian survey among women over 40. Maturitas.

[15] Kottner J, Audige L, Brorson S, Donner A, Gajewski BJ, Hróbjartsson A (2011). Guidelines for reporting reliability and agreement studies (GRRAS) were proposed. J Clin Epidemiol.

[16] Munro MG, Critchley HO, Fraser IS, FIGO Menstrual Disorders Committee (2018). The two FIGO systems for normal and abnormal uterine bleeding symptoms and classification of causes of abnormal uterine bleeding in the reproductive years: 2018 revisions. Int J Gynaecol Obstet.

[17] Kingsberg SA, Krychman ML (2013). Resistance and barriers to local estrogen therapy in women with atrophic vaginitis. J Sex Med.

[18] Calleja-Agius J, Brincat MP (2015). The urogenital system and the menopause. Climacteric.

